# Architectural approach to evaluate the design and management of almond cultivars suitable for super high-density orchards

**DOI:** 10.3389/fpls.2024.1407862

**Published:** 2024-07-23

**Authors:** Francesco Maldera, Simone Pietro Garofalo, Salvatore Camposeo

**Affiliations:** ^1^ Department of Soil, Plant and Food Sciences, University of Bari Aldo Moro, Bari, Italy; ^2^ Agriculture and Environment Research Centre - Council for Agricultural Research and Economics (CREA-AA), Bari, Italy

**Keywords:** vigor, biometry, brachyblasts, shoots, Filippo Cea, Guara, Lauranne

## Abstract

**Introduction:**

The almond tree is a major global nut crop, and its production has surged dramatically in recent years. Super high-density (SHD) planting systems, designed to optimize resource efficiency and enhance precocity, have gained prominence in almond cultivation. A shift in cropping systems toward sustainable intensification (SI) pathways is imperative, and so maximizing branching density within the canopies of SHD trees is crucial to establish and maintain productive potential, especially for hedge-pruned trees. This study investigates the influence of different almond cultivars grafted onto a novel growth-controlling rootstock on tree architectural and growth parameters in a SHD orchard. This open field research provided valuable insights for the development and application of new tools and methods to increase productivity and sustainability in almond growing.

**Methods:**

Three cultivars (Lauranne^®^ Avijour, Guara Tuono, and Filippo Cea) were evaluated in Gravina in Puglia (BA) over a two-year period. Canopy growth parameters, such as canopy volume and trunk cross-sectional area, and architectural traits, like branching density, branching angle, number and length of subterminal shoots, and number of brachyblasts, were measured through qualitative and quantitative measurements.

**Results and discussion:**

Results revealed significant differences in tree height, canopy thickness, width, volume, and vigor among the cultivars. Architectural traits, including branch parameters, brachyblast parameters, and subterminal shoots, varied among the cultivars. Lauranne displayed a more compact well-distributed canopy and exhibited the lowest vigor. Filippo Cea showed the highest vigor and the greatest canopy volume. Tuono had a higher number of buds and bud density. The best ideotype for SHD orchards is a smaller tree, with high branching density and smaller trunk diameters, i.e. the vigor. Cv. Lauranne seemed to be the best cultivar, mostly with the lowest tree vigor of all the cultivars involved. These findings provide valuable insights for almond growers and breeders seeking to optimize orchard design and management for enhanced SHD orchards productivity and sustainability. Future research will explore the relationship between canopy architecture and yield parameters, considering different scion/rootstock combinations in different environmental conditions.

## 1 Introduction

The almond tree (*Prunus amygdalus* (L.) Batsch = *P. dulcis* (Mill.)) is a dominant global tree nut crop, accounting for 31% of total nut crop production and with 700 thousand hectares planted worldwide (Iglesias and Torrents, 2022). Its production has risen substantially worldwide, increasing by 60% in the last ten years (USDA, 2024). The almond tree is widespread throughout the Mediterranean basin and is part of the agricultural biodiversity and food production systems, especially in Italy and Spain (Fernández i Martí et al., 2009; Socias i Company et al., 2009; Alhajj Ali et al., 2023). Sustainability and efficiency are key factors in new agriculture, in which every cultivation technique must be innovated to improve the soil-plant-environment system and the efficient use of the input (e.g. irrigation water management and fertilizers) (Morugán-Coronado et al., 2020; Garofalo et al., 2024). A shift in cropping systems toward Sustainable Intensification (SI) pathways is imperative. SI is broadly defined as increasing agricultural production on existing land, reducing environmental impacts, and enhancing the provision of environmental services (Cano et al., 2023; FAO, 2024). The demand for innovation in fruit tree cultivation has led to the development of highly efficient and sustainable planting systems that move beyond the traditional open vase tree shape, already studied and applied to other species, such as olive, in which intensification from low to Super High-density (SHD) presented the greatest improvements, both for yield and Net Ecosystem Productivity (Mairech et al., 2020). These new agronomic models for almond production focus on growth-controlling rootstocks, enabling orchard intensification with narrow rows and canopies, thereby enhancing input efficiency, particularly labor and mechanization, while promoting orchard precocity (Ghelfi and Palmieri, 2015; Iglesias et al., 2021; Maldera et al., 2023). The SHD planting system, also known as edge or “sustainable and efficient systems” (SES), has gained popularity, primarily promoted by the Agromillora group nursery in Spain (Casanova-Gascón et al., 2019; Iglesias et al., 2021; Maldera et al., 2021a; Iglesias and Torrents, 2022). This new system has already been applied to other crops, such as olives, since 1994 (Tous et al., 2010; Connor et al., 2014), in which higher tree density per hectare and full mechanization of all cultivation operations are used. The system resulted in improved water use efficiency (Romero-Trigueros et al., 2019) and in decreased environmental footprints with respect to traditional cropping systems (Pellegrini et al., 2016; Camposeo et al., 2022). Maximizing branching density within the canopies of SHD trees is crucial to establish and maintain productive potential, especially for hedge-pruned trees (Rosati et al., 2013; Vivaldi et al., 2015; Lodolini et al., 2017; Montesinos et al., 2023).

To optimize light interception and achieve uniform crop yield, major temperate tree fruit industries have adopted higher densities in growing systems with smaller trees, which offer new possibilities for almonds (Thorp et al., 2021). Efficient light interception is critical for photosynthesis, and hence for all physiological processes of the plant; optimal tree architecture helps maximize the interception of sunlight, enhancing photosynthesis rates and overall tree growth (Maldera et al., 2021a, 2023). Tree architecture has been studied extensively for apple orchards. Rootstock effect was observed as a key factor in modifying the canopy architecture of these orchards (Tworkoski and Miller, 2007; Domi et al., 2012; Fallahi et al., 2018). Previous research has been also done on olive tree architecture and its relationship with micropropagation (Neri et al., 2020) or different planting systems. Rosati et al. (2013) observed that high branching and small diameters are important architectural characteristics to increase yield efficiency and affect the cultivar suitability for SHD olive orchards, while Lodolini et al. (2017) focused attention on the different varietal architectural characteristics of the canopy and primary branches in olive cultivars (Carella et al., 2022).

The management of almond trees significantly affects the distribution of shoots and leaf area as well as the mechanization of pruning. This technique, as observed in SES growing systems, can offer substantial benefits by ensuring a more consistent product through uniform pruning practices (Franzen and Hirst, 2016). Understanding how different scion/rootstock combinations respond to different planting systems is essential for optimizing orchard management. Moreover, selecting appropriate cultivars remains one of the most effective strategies for ensuring the agronomic and economic viability of high-density cropping systems (Vivaldi et al., 2015). A whole range of different cultivars could be used in SHD orchards, such as Guara Tuono (Dicenta et al., 2015; Felipe, 2017) and Lauranne^®^ Avijour (Grasselly et al., 1990; Legave et al., 1997), leading to different responses (Maldera et al., 2021b). A size-controlling rootstock allows the growth of any cultivar, including autochthonous ones like Filippo Cea, noted for its seed health benefits (Monastra et al., 1982; Summo et al., 2018). Considering the importance of scion-rootstock combinations in almond orchards, it becomes crucial to explore their influence on the architecture of almond trees within the context of SHD orchards (Montesinos et al., 2021a, 2021b, 2022). Although several studies have investigated pruning and canopy architecture in several fruit tree crops, including almonds (Negrón et al., 2013; Negrón et al., 2015), limited research has focused specifically on the effect of scion-rootstock combinations on tree architecture in SHD almond orchards (Montesinos et al., 2023). Cultivars, pruning practices, and row orientation influences the architectural development of almond trees, which in turn impacts light interception, yield potential, and overall orchard management (Maldera et al., 2021a, 2023; Negrón et al., 2013; Negrón et al., 2015).

This research aimed to fill this knowledge gap by examining how different almond cultivars grafted onto a new growth-controlling rootstock influenced tree architectural and growth parameters in a young SHD orchard. This open field research intended to provide valuable insights for development and application of new tools and methods for agricultural systems. It planned to help in optimizing the design and management of new almond orchards, contributing to increased productivity and sustainability in almond growing.

## 2 Materials and methods

### 2.1 Site and orchard

The two-year study was carried out from May 2021 to January 2023 in an irrigated SHD almond orchard, with tree spacing of 3.8 m x 1.2 m (2,190 trees ha^-1^), planted in April 2020 in Gravina in Puglia (Southern Italy; 40° 53’ 02.2” N; 16° 18’ 40.3” E; 400 m a.s.l.). The soil was classified as a loam soil with a pH of 8.0, 2% active carbonates, and an organic matter content of 1.4%. Three different cultivars were evaluated: Lauranne^®^ Avijour, Tuono (=Guara), and Filippo Cea. All cultivars were grafted onto Rootpac-20^®^, a new patented size-controlling rootstock (*P. besseyi* x *P. cerasifera*), obtained by the Agromillora breeding program (Iglesias et al., 2021). Three experimental plots of 1200 m^2^ each, separated by approximately 30 m, were established, one for each cultivar. Each plot consisted of five rows, in which the three central rows were taken as the sampling area.

### 2.2 Cultural practices

Orchard management was performed through the best common practices diffused in the area, following the integrated farming method. Drip lines, suspended at 0.7 m over the soil, were used, and seasonal average volumes of irrigation of approximately 5000 m^3^ ha^-1^ were applied. Every year, after leaf fall (November-December), mechanical topping, hedging, and trimming were carried out to control canopy growth. The first harvest was operated in summer 2022, at the third year after planting, by a continuous over-the-row harvester machine (New Holland).

### 2.3 Canopy growth parameters

Biometric parameters were measured on four different dates (June 2021, January 2022, May 2022, and January 2023), in fifty trees per row, consisting of 150 trees measured. The measured parameters were tree height (TH, cm), tree width (canopy in the row, TW, cm), tree thickness (canopy towards the inter-row, TT, cm), and trunk diameter (TD, cm) at half the height from ground to first branches. Trunk cross-sectional area (TCSA, cm^2^; Equation 1) was calculated as


(1) $$\text{TCSA}=\pi \left({\text{TD}}^{2}\right)/4$$

Canopy volume (CV, m^3^; Equation 2) was always calculated considering the canopy as a parallelepiped shape, with 0.4m subtracted to TH, representing the distance of the first branches from the ground:


(2) CV=(TH−0.4) ×TW ×TT

### 2.4 Architectural parameters

Data collection was carried out on two different dates (January 2022 and January 2023). A whole range of architectural traits were measured on field.

#### 2.4.1 Screening index

A screening index (IS) was given to fifty trees per row, leading to 150 trees for cultivar, following Neri et al. (2020); a value was given to the trees, ranging from low branching and more pore spaces in the row (1), to high branching and less pore spaces (5). Therefore, we calculated the frequency (%) of each class.

#### 2.4.2 Qualitative traits

Following the work of Montesinos et al. (2023), a wide range of architectural parameters were evaluated. For fifteen trees per cultivar, a total of 10 parameters were evaluated from different categories: branches habit, brachyblasts (or dard) habit, and sub terminal shoots habit (**Table 1**). Per each parameter, a value from 1 to 5 was given that best represented the tree.

**Table 1:** Qualitative traits and parameters evaluated, with extreme class values (1-5).

Trait	Para meter	Abbre viation	Meas. unit	1		2	3	4	5	
**Branches** **habit**	Number	Br_N	n	Few	≤1	1-2	2-3	3-4	>4	Many
	Diameter	Br_D	mm	Reduced	≤10	10-20	20-30	30-40	>40	Wide
	Length	Br_L	cm	Short	≤10	10-20	20-30	30-40	>40	Long
	Angle	Br_A	UPOV Scale	Open	Drooping	spreading to drooping	Spreading	upright to spreading	Upright	Close
**Brachyblasts** **habit**	Number	Bb_N	n	Few	≤10	10-20	20-30	30-40	>40	Many
	Diameter	Bb_D	mm	Reduced	≤5	5-10	10-15	15-20	>20	Wide
	Length	Bb_L	mm	Short	≤1	1-2	2-3	3-4	>4	Long
**Sub-terminal** **Shoots habit**	Number	Su_N	n	Few	≤1	2-3	3-4	4-6	>6	Many
	Diameter	Su_D	mm	Reduced	≤4	4-8	8-12	12-15	>15	Wide
	Length	Su_L	cm	Short	≤10	10-20	20-30	30-40	>40	Long

#### 2.4.3 Quantitative traits

Two (one-year) shoots per tree were taken in order to observe shoot length (SL, cm), the total number of buds (TB, n), wood and flower buds’ percentages (WB and FB, %), and the bud density on the shoot (BD, n cm^-1^).

### 2.5 Statistical analysis

Normality test and homoscedasticity test were performed for each set of quantitative data considered in this study using Shapiro-Wilk’s test and Levene’s test, respectively. After noncompliance with the assumptions to perform an ANOVA test was detected, Kruskal-Wallis rank sum test (KW test) was performed to compare the medians, using Dunn’s *post-hoc* comparison test (Scheff, 2016). Spearman correlation test was performed among the different parameters under investigation for the three cultivars. Fisher’s test has been used to compare trait distributions among the cultivars. The statistical analyses were carried out with RStudio for Windows (Rstudio, PBC, Boston, MA; 2022.12.0 + 353); probability levels used were *p<* 0.05 (*), *p<* 0.01 (**), *p<* 0.001 (***) and *p<* 0.0001 (****).

## 3 Results

**Figure 1:**
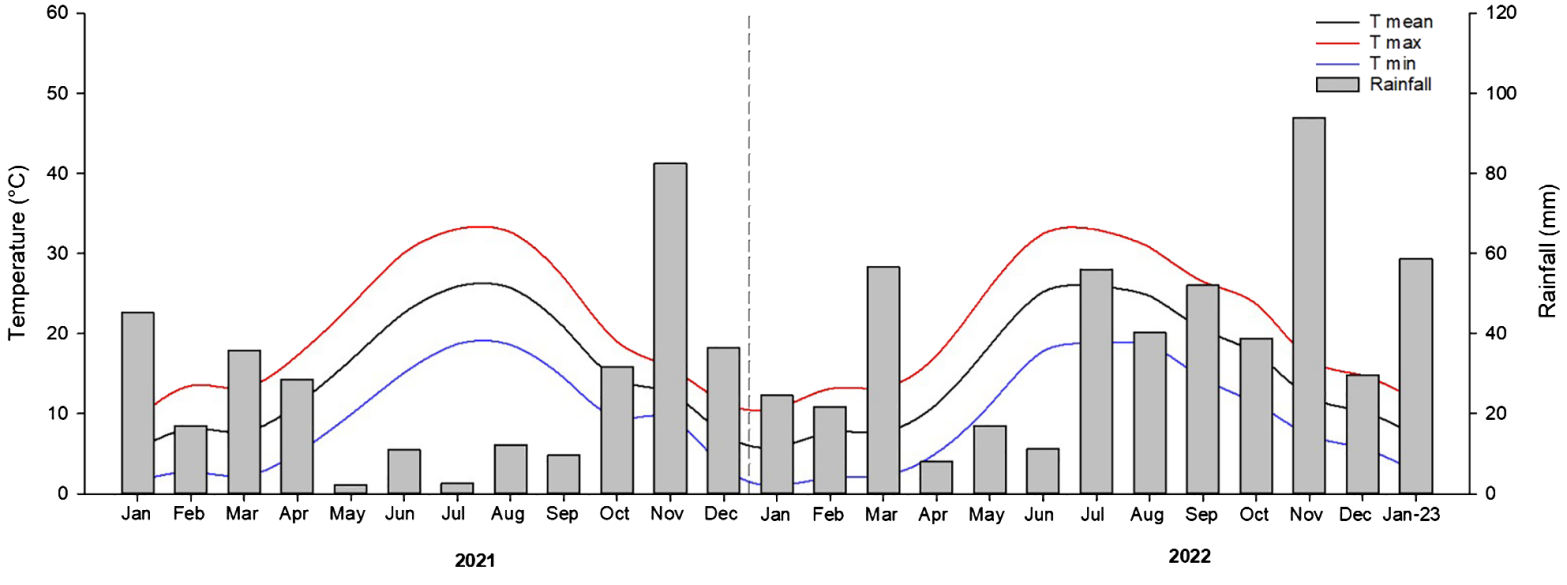
Monthly rainfall and trend of temperatures recorded in the experimental almond orchard from January 2021 to January 2023. Source: Protezione civile regione Puglia. Bollettini meteorologici regionali mensili.

**Figure 1** showed the typical mediterranean climate, characterized by an average annual rainfall of 380 mm/year; the lowest value of monthly rainfall was observed in May for 2021 (2.2 mm) and April for 2022 (8.0 mm), while the highest value was rainfall was in November for both years (82.6 and 93.9 mm respectively).

### 3.1 Canopy growth parameters

**Figure 2:**
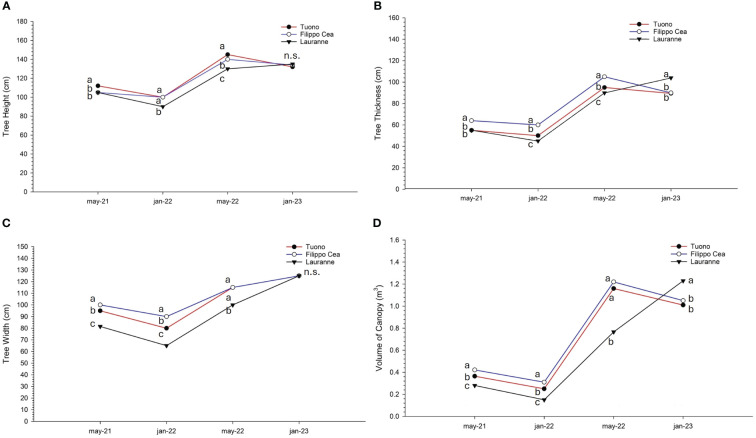
Median of tree height **(A)**, thickness **(B)**, width **(C)**, and volume of the canopy **(D)** of the cultivars. Tuono (red), Filippo Cea (blue), and Lauranne (black). Letters indicate significant differences among the three cultivars (Tuono, Filippo Cea, Lauranne) with median at *p*< 0.05.

Cultivar deeply influenced canopy size (**Figure 2**). On the first sampling session cv. Tuono showed higher TH than both Filippo Cea and Lauranne (112, 105 and 105 cm, respectively), remaining the highest until the last measurement date (**Figure 2A**). Lauranne showed the lowest values since the beginning, reaching the other two cultivars only at the last measurement, in January 2023. Filippo Cea exhibited medium values, ever keeping a medium size. A different situation was observed for TT (**Figure 2B**): the cultivar that showed the greatest canopy thickness was Filippo Cea, followed by cvs. Tuono and Lauranne. This situation clearly changed during the last measurement, in which cv. Lauranne statistically exceeded the other two cultivars (104 cm for Lauranne, 90 cm and 89.5 cm for Tuono and Filippo Cea, respectively).

TW showed a very similar trend to the one observed for TT (**Figure 2C**). The highest values were observed for cv. Filippo Cea, especially in the first two measurements, while from the third measurement cv. Tuono also reached the same width (115 cm for both cultivars). The cultivar with the lowest TW was found to be Lauranne, but it reached the same value during the last measurement (125 cm for all cultivars).

Consequentially, the cultivar with the highest canopy volume was found to be Filippo Cea (**Figure 2D**), statistically equal to cv. Tuono (0.6 and 0.34 m^3^). Moreover, for the first three measurements, cv. Lauranne showed much less volume than the other two cultivars, particularly in the May 2022 measurement (0.77 m^3^ compared to 1.16 and 1.22 m^3^ of cvs. Tuono and Filippo Cea, respectively). Just as observed for TH and TT, in January 2023 cv. Lauranne showed the highest VC value (1.25 m^3^).

**Figure 3:**
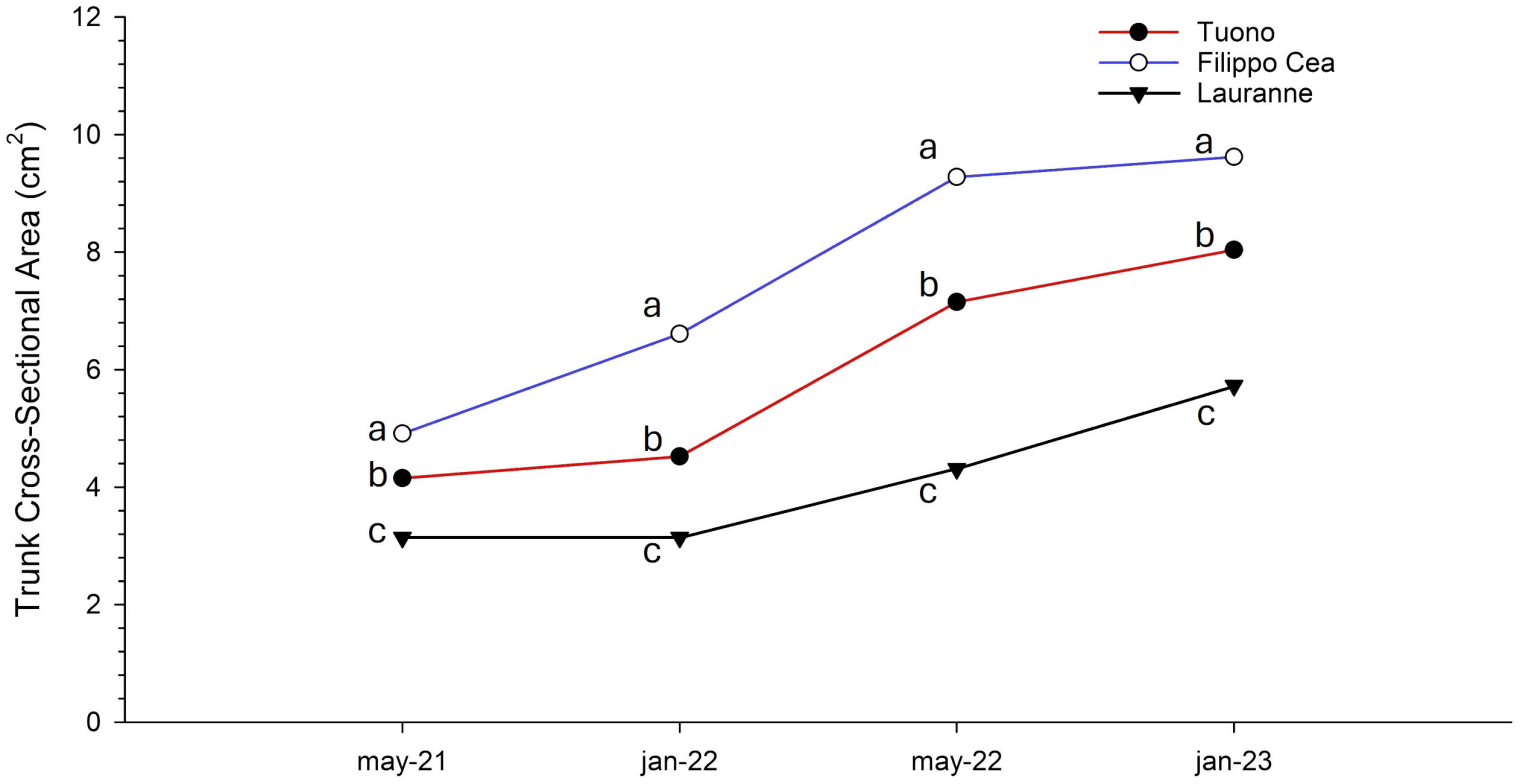
Median of trunk cross-sectional area (TCSA) of the cultivars Tuono (red), Filippo Cea (blue), and Lauranne (black). Letters indicate significant differences among the three cultivars (Tuono, Filippo Cea, and Lauranne) with median at *p*< 0.05.

Finally, the three cultivars were completely separated in terms of vigour, expressed as TCSA (**Figure 3**). An increasing trend for all three cultivars was observed, with cv. Filippo Cea consistently and significantly the most vigorous (reaching a value of 9.62 cm^2^ in January 2023) and cv. Lauranne the least vigorous (with the lowest value of 5.12 cm^2^ in in January 2023).

### 3.2 Architectural parameters

#### 3.2.1 Screening index

**Figure 4:**
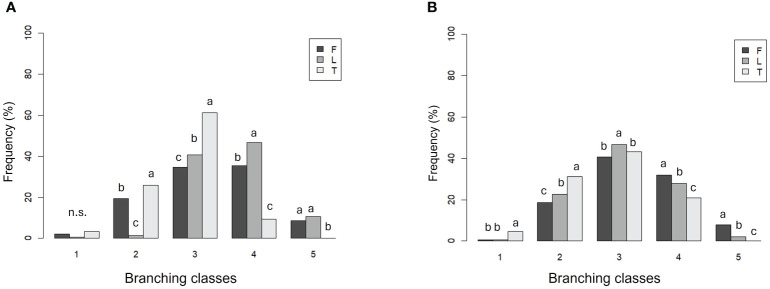
Screening index classes frequency (%) of cultivars Tuono (T), Filippo Cea (F), and Lauranne (L) in 2022 **(A)** and 2023 **(B)**. Class 1 (low branching and more pore spaces in the row) to class 5 (high branching and less pore spaces).

IS values varied differently depending on the cultivar (**Figure 4**). In 2022, cv. Tuono showed the highest percentage in class 3 followed by class 2 (63% and 21%, respectively), while no tree was assigned in class 5 (**Figure 4A**). On the contrary, cv. Filippo Cea showed the highest percentage in class 4 (40%), followed by class 3 and 5 (39% and 6%, respectively). Finally, cv. Lauranne showed the highest IS percentage for class 4 (45%), and nearly no tree was assigned to classes 2 and 1.

A similar but less pronounced situation was observed for 2023 (**Figure 4B**). Tuono showed higher total percentage of trees in the lowest classes (2 and 3), while Filippo Cea and Lauranne showed similar results, with the highest percentages in class 3 and the remaining trees assigned mostly to classes 4 and 5.

#### 3.2.2 Qualitative traits

The architectural parameters of branches showed relevant differences among the three cultivars (**Table 2**). Br_N showed slight differences among the different cultivars: classes 2 and 3 were the most present in all three cultivars, but for cv. Tuono indicated more than 90% of the trees. Cultivars Filippo Cea and Lauranne showed more distributed Br_N, with higher values in class 1 but also in class 4, in which cv. Filippo Cea revealed the highest value (13.3%). Br_D was hardly influenced by the cultivars. Tuono and Lauranne showed the same distribution, while cv. Filippo Cea presented different class distributions: while the first two cultivars concentrated almost exclusively on trees in 2 and 3 categories, with higher values in class 2, cv. Filippo Cea showed higher values in class 3, more than 60.0%, but also with 17% of trees in class 4. Branch Length (Br_L) showed a different response than Br_D. In fact, while a higher percentage of trees were allocated to class 2 in cv. Tuono, cultivars Filippo Cea and Lauranne presented higher values in class 3, with a medium length of the branches. No relevant differences were observed in class 4, in which the highest values were recorded in cv. Lauranne (10%). Br_A was strictly related to the cultivar as well. While cv. Filippo Cea presented more trees in class 1 and 2 (10 and 50%, respectively), cv. Tuono showed an opposite behavior, with a higher percentage of trees in classes 3 and 5 (43.3 and 16.7%, respectively). Lauranne, on the other hand, showed a homogeneous distribution in the classes, with peaks recorded in classes 3 and 4 (33.3% in both classes).

**Table 2:** Frequency distribution (classes) of qualitative architectural parameters of the branches for cultivars Tuono, Filippo Cea, and Lauranne.

Parameter	Cultivar	Classes (%)									
		1		2		3		4		5	
**Br_N**	Tuono	6.7	n.s.	46.7	n.s.	46.7	n.s.	0	n.s.	0	
	Filippo Cea	13.3		40.0		33.3		13.3		0	
	Lauranne	16.7		36.7		40.0		6.7		0	
**Br_D**	Tuono	0	n.s.	50.0	**	43.3	n.s.	6.7	n.s.	0	
	Filippo Cea	3.3		16.7		63.3		16.7		0	
	Lauranne	0		50.0		43.3		6.7		0	
**Br_L**	Tuono	0	n.s.	53.3	n.s.	40.0	n.s.	6.7	n.s.	0	
	Filippo Cea	0		30.0		63.3		6.7		0	
	Lauranne	0		30.0		60.0		10.0		0	
**Br_A**	Tuono	3.3	n.s.	13.3	**	43.3	n.s.	23.3	n.s.	16.7	n.s.
	Filippo Cea	10.0		50.0		20.0		16.7		3.3	
	Lauranne	3.3		16.7		33.3		33.3		13.3	

Br_N, Branches number; Br_D, Branches diameter; Br_L, Branches length; Br_A, Branches angle. Class 1 (low branches number, diameter, length, and open angle) to class 5 (high branches number, diameter, length, and close angle). n.s., not significant; ** Stand for significant at P<0.01.

Brachyblasts parameters showed interesting patterns in the different cultivars (**Table 3**). The most important differences were observed for Bb_N (i.e. number of brachyblasts), in which all the cultivars showed a lower number of trees in classes 4 and 5. While cv. Tuono presented 80% of the trees in classes 2 and 3 (50 and 30%, respectively), cvs. Lauranne and Filippo Cea showed more distributed values, but mainly concentrated in classes 1 and 2. Bb_L and Bb_D presented nearly the same allocation in classes: cvs. Filippo Cea and Lauranne were mostly allocated in class 1, while higher percentages were registered in class 2 for Bb_D (43.3%) and in classes 2 and 3 for Bb_L (23.3 and 13.3, respectively).

**Table 3:** Frequency distribution (classes) of qualitative architectural parameters of the brachyblasts for cultivars Tuono, Filippo Cea, and Lauranne.

Parameter	Cultivar	Classes (%)								
		1		2		3		4		5
**Bb_N**	Tuono	16.7	*	50.0	n.s.	30.0	n.s.	3.3	n.s.	0
	Filippo Cea	36.7		33.3		23.3		6.7		0
	Lauranne	50.0		26.7		13.3		10.0		0
**Bb_L**	Tuono	63.3	**	23.3	**	13.3	n.s.	0		0
	Filippo Cea	96.7		0		3.3		0		0
	Lauranne	76.7		23.3		0		0		0
**Bb_D**	Tuono	56.7	*	43.3	**	0	n.s.	0		0
	Filippo Cea	90		10		0		0		0
	Lauranne	80		16.7		3.3		0		0

Bb_N, Brachyblasts’ number; Bb_L, Brachyblasts’ length; Bb_D, Brachyblasts’ diameter. Class 1 (low brachyblasts number, length, and diameter) to class 5 (high brachyblasts number, length, and diameter). n.s., not significant; * Stand for significant at P<0.05., ** Stand for significant at P<0.01.

Sub terminal shoot parameters were strongly influenced by the cultivar (**Table 4**). Cultivar Tuono showed the lowest number of Su_N, with nearly 90% of the trees in classes 1 and 2 (36.7 and 50%, respectively). A different behavior was observed for cultivars Filippo Cea and Lauranne: while the former had more trees in classes 2 and 3 (33.3 and 50%, respectively), the latter also had 20% of trees in class 4, revealing a higher ramification of the canopies. Su_D showed different patterns for the three cultivars. While cv. Tuono presented the highest percentages in classes 3 and 4, the opposite was observed in classes 1 and 2. The highest number of cv. Lauranne trees belonged to class 2 (70%); cv. Filippo Cea registered a higher number of trees in class 1 (43.3%), but the rest of the trees were found to be equally distributed among the other classes. For Su_L, opposite patterns were observed for cultivars Tuono and Filippo Cea: while the former showed higher percentages of trees in the higher classes, with 40% in classes 4 and 5, the latter had nearly 35% in the lowest ones (16.7% in both class 1 and 2). Lauranne showed mostly mid values, with slightly higher values in class 2 than class 4 (33.3 and 20%, respectively).

**Table 4:** Frequency distribution (classes) of qualitative architectural parameters of the subterminal shoots for cultivars Tuono, Filippo Cea, and Lauranne.

Parameter	Cultivar	Classes (%)									
		1		2		3		4		5	
**Su_N**	Tuono	36.7	*	50.0	n.s.	13.3	**	0	*	0	
	Filippo Cea	6.7		33.3		50.0		10.0		0	
	Lauranne	16.7		36.7		26.7		20.0		0	
**Su_D**	Tuono	0	***	50.0	***	30.0	*	20.0	*	0	
	Filippo Cea	43.3		20.0		30.0		6.7		0	
	Lauranne	23.3		70.0		6.7		0		0	
**Su_L**	Tuono	0	**	16.7	n.s.	43.3	n.s.	30.0	n.s.	10.0	n.s.
	Filippo Cea	16.7		16.7		40.0		26.7		0	
	Lauranne	0		33.3		46.7		20.0		0	

Su_N, Subterminal shoots number; Su_D, Subterminal shoots diameter; Su_L, Subterminal shoots length. Class 1 (low subterminals number, diameter, and length) to class 5 (high subterminals number, diameter, and length). n.s., not significant; * Stand for significant at P<0.05, ** Stand for significant at P<0.01, *** Stand for significant at P<0.001.

#### 3.2.3 Quantitative traits

**Figure 5:**
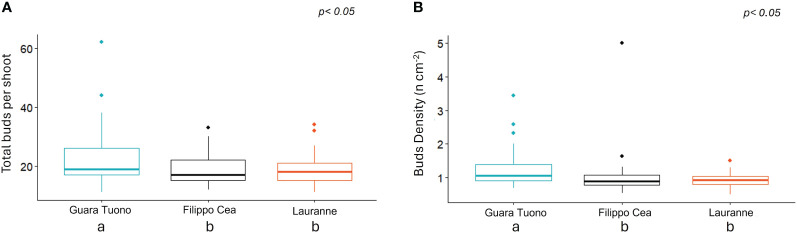
Total buds per shoot **(A)** and bud density **(B)** for cultivars Tuono, Filippo Cea, and Lauranne. Letters denote statistical differences among cultivars at *p*< 0.05.

No statistical differences were observed for shoot length or wood and flower buds’ percentages. However, the cultivar influenced both the total number of buds and bud density (**Figure 5**). In particular, cv. Tuono exhibited higher and statistically significant values compared to the other two cultivars, both in terms of the total number of buds (22.5 for Tuono and 18.8 for Filippo Cea and Lauranne, respectively, **Figure 5A**) and bud density (1.23, 1.00, and 0.93 buds cm^-2^ for cultivars Tuono, Filippo Cea, and Lauranne, respectively, **Figure 5B**).

**Table 5** reports the most significant correlation coefficients that could comprehensively explain the differences among the cultivars. These correlations were found to be highly significant (p-value< 0.01). The correlation between SL and WB was found to be significantly relevant for both cultivars Filippo Cea and Lauranne, with very similar values observed between them. The same identical pattern was observed for the SL-TB pair, although a higher correlation was observed for the second cultivar (0.62). No correlation was observed for cv. Tuono in both pairs. Different behaviors were observed among the cultivars regarding the FB-TB and WB-TB correlations. While a higher correlation was observed for Lauranne in the former (0.63 compared to 0.5 and 0.51), the latter correlation showed opposite values, with higher correlations for the other two cultivars (0.85 and 0.83 for Filippo Cea and Tuono, respectively, compared to 0.73 for Lauranne). Bud density was found to be statistically significant in correlations with flower buds (FB-BD) and total buds (TB-BD). In particular, a statistically significant correlation was observed in both pairs for cultivars Tuono and Lauranne, with greater differences observed for BD-TB, where Tuono showed a higher correlation. In both cases, no statistically significant correlation was observed for Filippo Cea.

**Table 5:** Most significant Spearman correlations coefficients (p< 0.05) among the quantitative traits for cultivars Tuono, Filippo Cea, and Lauranne.

Cultivar	SL-WB	SL-TB	FB-TB	WB-TB	FB-BD	BD-TB
**TUONO**	–	–	0.50	0.83	0.46	0.83
**FILIPPO CEA**	0.55	0.55	0.51	0.85	–	–
**LAURANNE**	0.53	0.62	0.63	0.73	0.44	0.51

SL, Shoot Length; WB, Wood buds; TB, Total number of buds; FB, Flower buds; BD, Buds density.

## 4 Discussion

The results of this study show that the cultivar has a significant impact on the canopy growth (**Figure 2**). The cultivar Guara Tuono consistently had the highest tree height (TH) out of the three cultivars. This difference in TH was maintained throughout the measurement period. Similarly, Tuono had the greatest canopy thickness (TT), followed by Filippo Cea and Lauranne. However, in the last measurement, Lauranne surpassed the other two cultivars in TT.

Canopy width (TW) showed similar patterns to TT, with Filippo Cea having the highest values in the first two measurements. However, from the third measurement, Tuono also reached the same width as Filippo Cea. Lauranne had the lowest TW initially but reached the same value as the other cultivars in the last measurement. This behavior was different from previous results, in which Lauranne Avijour showed higher canopy dimensions (Maldera et al., 2021b). The cultivar Filippo Cea had the highest canopy volume (VC), followed by Tuono, while Lauranne had the lowest volume. This trend in VC was consistent with the patterns observed in TH and TT. These results were surely influenced by mechanical pruning operations, equally performed throughout the orchard, that imposed certain dimensions to the three cultivars. It is already known that mechanical pruning is imperative in SHD orchards to fill and reduce the number of holes in the canopy and the costs of the operation (Fumey et al., 2011; Ferguson et al., 2012; Negrón et al., 2015; Vivaldi et al., 2015).

In terms of tree vigor, Filippo Cea consistently showed the highest values, indicating it was the most vigorous cultivar, while Lauranne exhibited the lowest vigor (**Figure 3**). These data are in contrast with Maldera et al., (2021b), in which Lauranne showed more vigorous trees than Guara Tuono, which is in line with Montesinos et al. (2023) where Lauranne grafted on Rootpac^®^ 20 showed low vigor and lower canopy dimensions. Ben Yahmed et al. (2016) observed no differences in terms of vigor between Lauranne and Guara. Ben Yahmed et al. (2022) observed an opposite behavior in the same region, with Lauranne more vigorous than Guara and Tuono. In this case, as for other species, differences in pedoclimatic conditions and scion-rootstock combinations could lead to these responses (Warner, 1991; Weibel et al., 2003; Gullo et al., 2014; Lodolini et al., 2017; Fallahi et al., 2018; Ben Yahmed et al., 2022).

Canopy architecture plays a key role in the development of trees structure (Asai et al., 1996; Lauri et al., 2009, 2011; Jiménez-Brenes et al., 2017). The screening index (IS) revealed that Guara Tuono had the highest percentage of trees in class 3, followed by class 2 (**Figure 4**). Filippo Cea and Lauranne had the highest percentage in class 4. These findings seem promising, considering a different behavior of Guara Tuono: this cultivar struggles to close the production wall, having a less open shape, with more gaps and less branching. The IS played an important role, showing a not well-adapted canopy structure of cv. Tuono. Montesinos et al. (2023) observed the same situation, with a better ramification of Lauranne, in all the different scion-rootstock combinations.

The branches parameters showed some differences among the cultivars. Guara Tuono had the highest percentage of trees in classes 2 and 3 for branch number (Br_N) (**Table 2**). Filippo Cea had higher values in class 4 for branch diameter (Br_D), while Guara Tuono and Lauranne had similar distributions. Branch length (Br_L) showed higher values in class 3 for Filippo Cea and Lauranne, while Guara Tuono had a higher percentage in class 2. Branch angle (Br_A) differed among the cultivars, with Filippo Cea having more trees in classes 1 and 2, and Guara Tuono having more trees in classes 3 and 5. Montesinos et al. (2023) showed exactly the same values: Guara Tuono exhibited lower and shorter branches than Lauranne Avijour, while no comparisons can be made with Filippo Cea, because no bibliography references have been done yet on this cultivar.

For brachyblast parameters, all cultivars had fewer trees in classes 4 and 5 for brachyblast number (Bb_N) (**Table 3**). Tuono had the highest percentage of trees in classes 2 and 3, while Lauranne and Filippo Cea had more distributed values, mainly in classes 1 and 2. Brachyblast length (Bb_L) and Brachyblast diameter (Bb_D) showed similar distributions among the cultivars, with higher values in class 1 for both parameters. These results are in contrast with previous ones, in which no relevant differences were observed (Montesinos et al., 2023).

Subterminal parameters were influenced by the cultivar. Tuono had the lowest number of subterminals (Su_N), mainly in classes 1 and 2. Filippo Cea had higher percentages in classes 2 and 3, while Lauranne had 20% of trees in class 4, indicating a higher ramification of the canopies (**Table 4**). Subterminal diameter (Su_D) showed higher percentages in classes 3 and 4 for Tuono, while Lauranne had the most trees in class 2. For subterminal length (Su_L), Tuono had higher percentages in the higher classes, while Filippo Cea had more trees in the lower classes. Lauranne showed mid-values, with slightly higher values in class 2 than class 4. These findings are totally in line with IS data, observing a reiterated behavior of the Lauranne and Filippo Cea to be more branched, while Tuono is the one with the lowest and longest branches.

No statistical differences were observed for branch length, flower buds, and wood buds among the cultivars. However, the cultivar Tuono had higher values for both the total number of buds and bud density than Filippo Cea and Lauranne.

After the observation of all the examined parameters, an overview architectural model of the three cultivars can be done.

Tuono had lower growth than Filippo Cea but higher than Lauranne. It showed the lowest number of branches and, consequently, the highest number of pore spaces, leaving more holes in the hedgerow. These parameters are strictly related to its branching habit. Brachyblasts are more frequent with respect to the other cultivars but longer and bigger. Subterminals are the fewest, the biggest, and the longest, making more pruning necessary during the year. With the same length, the highest number of buds and, consequentially, bud density have been recorded, also confirmed by the correlations, in which the total number of buds was highly correlated to the number of wood buds and bud density.

Filippo Cea showed the highest growth and, more importantly, the highest vigor. It is characterized by the best screening index, due to an excellent distribution and occupation of space. This is related to its branching habit, which is characterized by more open branches, even if they are lower in number and with medium length and diameter. Average number and lowest diameter and length values characterized the brachyblasts. Subterminals are characterized by a medium number but the lowest diameter and length. Lower buds per shoot and bud density were observed, with the highest correlation between wood buds and total bud number.

Lauranne exhibited the lowest tree vigor. A medium screening index was observed, with a good distribution of the branches in the space. High number, average diameter, highest length, and a different angle characterized the branches. The fewest number of brachyblasts, with an average diameter and length, were observed. Subterminal shoots were the most numerous and showed the smallest diameter. A good number of buds per shoot and an average bud density were also observed. Interesting correlations were also observed between total buds and wood, flower buds, and shoot length.

In an SHD orchard, the best ideotype is a small tree with high branching density and small trunk diameters (Rosati et al., 2013; Vivaldi et al., 2015; Lodolini et al., 2017; Thorp et al., 2021; Montesinos et al., 2023). The cv. Lauranne seemed to fill the different spaces in the canopy better and faster, mostly with the lowest tree vigor of all the cultivars involved. This scion/rootstock combination could help to reach the goal of sustainable intensification, which has been the main pursuit of recent years (Cano et al., 2023).

## 5 Conclusion

The architectural approach can help growers to reach the goal of a full trained hedgerow better and faster, leading to the goal of a Sustainable and Efficient System (SES). The cultivar choice strongly influenced canopy dimensions, vigor, and architectural traits. Different cultivars exhibited variations in tree height, canopy thickness, width, volume, vigor, and various architectural traits such as branch parameters, brachyblasts parameters, and subterminal shoots parameters. Cv. Lauranne showed the best results in growth and in distribution of the branches, while Tuono seemed to be the worst distributed one. Cv. Filippo Cea showed the highest volume of the canopy, showing an intermediate behavior for the other architectural parameters. These findings provide valuable information for the development and application of tools and methods for agricultural systems design, assessment, and management. This work can help almond growers and breeders in selecting cultivars based on desired architectural traits and vigor, enhancing the productivity and sustainability of SHD orchards. Future research will explore the relationship between canopy architecture and yield parameters, considering different scion/rootstock combinations by using new size-controlling rootstocks like Pilowred^®^ or Rootpac-R^®^.

## Data availability statement

The raw data supporting the conclusions of this article will be made available by the authors, without undue reservation.

## Author contributions

FM: Conceptualization, Investigation, Methodology, Project administration, Visualization, Writing – original draft, Writing – review & editing. SPG: Data curation, Formal analysis, Methodology, Software, Writing – original draft, Writing – review & editing. SC: Funding acquisition, Resources, Supervision, Validation, Writing – review & editing.
